# Investigation of the relationship between serum irisin level in the idiopathic restless legs syndrome: Could be a marker independent of physical activity?

**DOI:** 10.1002/brb3.3100

**Published:** 2023-05-28

**Authors:** Sena Boncuk Ulaş, Türkan Acar, Bilgehan Atılgan Acar, Semra Alaçam Köksal, Yeşim Güzey Aras, Aybala Neslihan Alagöz, Sıdıka Sinem Gül, Nimet Uçaroğlu Can, Mehmet Köroğlu

**Affiliations:** ^1^ Department of Neurology Keşan State Hospital Edirne Turkey; ^2^ Department of Neurology Sakarya University Faculty of Medicine Sakarya Turkey; ^3^ Department of Neurology Kocaeli İzmit SEKA State Hospital Kocaeli Turkey; ^4^ Department of Neurology Kocaeli University Faculty of Medicine Kocaeli Turkey; ^5^ Department of Neurology Yenikent State Hospital Sakarya Turkey; ^6^ Department of Neurology Sakarya University Training and Research Hospital Sakarya Turkey; ^7^ Department of Microbiology Sakarya University Faculty of Medicine Sakarya Turkey

**Keywords:** irisin, physical activity, restless legs syndrome

## Abstract

**Introduction:**

Restless legs syndrome (RLS) is a common but underdiagnosed neurological syndrome. It is characterized by the feeling of discomfort and desire to move, especially in the lower extremities, which often occurs at night, and the cure or relief of symptoms with movement. Irisin is a hormonelike polypeptide that was first identified in 2012, weighs 22 kDa, consists of 163 amino acids, and is mainly synthesized in muscle. Its synthesis increases with exercise. Here in this study, we planned to investigate the relationship among serum irisin level, physical activity, lipid profile, and RLS.

**Material and methods:**

A total of 35 patients with idiopathic RLS and 35 volunteers were included in the study. Then, venous blood was taken from the participants in the morning after 12 h of night fasting.

**Results:**

The mean value of serum irisin level was 16.9 ± 14.1 ng/mL in the case group and 5.1 ± 5.9 ng/mL in the control group, which was statistically quite significant (*p* < .001). A significant efficiency (under the curve area 0.886 [0.804–0.967]) of irisin value was observed in the differentiation of patients in the case and control groups.

**Discussion:**

Serum irisin level was significantly higher in the case group than in the control group. In conclusion, we suggest that irisin may play a role in the pathophysiology of RLS independently of the intensity and duration of physical activity and anthropometric data, such as body weight, body mass index, and waist/hip ratio.

## Graphical abstract

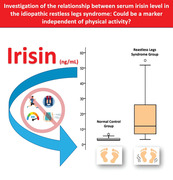

## 1 INTRODUCTION

Restless legs syndrome (RLS), also known as Willis–Ekbom disease, was first described in 1672 and is a common but underdiagnosed neurological syndrome (Gossard et al., 2021). It is characterized by the feeling of discomfort and desire to move, especially in the lower extremities, which often occurs at night, and the cure or relief of symptoms with movement. More rarely, symptoms may occur in other parts of the body and during the day (Klingelhoefer et al., 2016; Manconi et al., 2021). RLS can be seen as idiopathic (primary) or secondary. There is no etiological factor in idiopathic RLS, but genetic factors could be influential. Secondary RLS occurs in conditions, such as iron deficiency anemia, kidney failure, and pregnancy (Klingelhoefer et al., 2016). Although centuries have passed since the first recognition of RLS, its pathophysiology is still unclear. Brain iron metabolism, dysfunction in dopaminergic pathways, and glutamate and adenosine‐related changes are held responsible for the pathophysiology (Gonzalez‐Latapi & Malkani, 2019; Manconi et al., 2021).

Irisin is a hormonelike polypeptide that was first identified in 2012, weighs 22 kDa, consists of 163 amino acids, and is mainly synthesized in muscle (Cheng et al., 2021). Its synthesis increases with exercise. Irisin is formed by the degradation of a transmembrane protein called fibronectin type III domain‐containing protein 5 (FNDC5) by enzymatic activity. After irisin is synthesized in the muscle, it passes into the blood and is transported to the adipose tissue. It plays a role in the conversion of subcutaneous white adipose tissue into brown adipose tissue, regulation of insulin metabolism, reduction of insulin resistance, and regulation of energy metabolism (Chen et al., 2016; Huang et al., 2020). Irisin is also responsible for cholesterol metabolism. With the increase in irisin secretion, total cholesterol synthesis decreases, while hepatobiliary cholesterol secretion and fecal cholesterol excretion increase (Cheng et al., 2021).

Recent studies have shown that the FNDC5/Irisin complex stimulates the release of brain‐derived neurotrophic factors (BDNF), especially in the hippocampus, and plays a role in neuroplasticity (Jodeiri Farshbaf & Alviña, 2021; Lourenco et al., 2019). For this reason, recent studies examining the relationship between Alzheimer's disease, cognitive functions, and Irisin are seen in the literature (Lourenco et al., 2019). However, as far as we know, a study has not been examined before to investigate the relationship between the Irisin molecule, released from muscle with exercise, and RLS, a disease in which symptom relief is seen with movement. Here in this study, we planned to investigate the relationship among serum irisin level, physical activity, lipid profile, and RLS.

## 2 MATERIAL AND METHODS

Patients who applied to Sakarya University Training and Research Hospital Neurology Clinic Movement Disorders Outpatient Clinic were analyzed retrospectively between November 2016 and April 2017. Patients diagnosed with idiopathic RLS according to the consensus criteria of International Restless Legs Syndrome Study Group (IRLSSG) published in 2014 were evaluated (Allen et al., 2014). Patients were included in the study before starting RLS treatment at their first apply to outpatient clinic. Those under the age of 18 and over the age of 70, history of anemia, diabetes mellitus, peripheral neuropathy, peripheral artery disease and chronic renal failure, pregnancy, and patients with physical/mental disabilities were excluded. A total of 35 patients with idiopathic RLS were included in the study. Volunteers from our clinic in whom neurological disorders had been ruled out conformed the control group. Informed consent was obtained from all participants. Ethics committee approval was obtained from our university before starting the study.

Age, gender, educational status, occupation, and smoking status of all participants were noted as sociodemographic data. Height, body weight, body mass index (BMI), waist circumference, hip circumference, and waist/hip ratio were calculated and recorded as anthropometric measurements. Based on the cut‐off values of waist circumference and waist/hip ratio for the Asian population published by the World Health Organization in 2008, the participants have divided into three groups according to waist circumference; no risk, risky, and very risky, and divided into two groups according to waist/hip ratio; no risk and risky in terms of metabolic complications (World Health Organisation (WHO), 2008). Accordingly, the waist circumference of above 94 cm in men and above 80 cm in women is risky, above 102 cm in men and 88 cm in women is very risky; waist/hip ratio ≥0.90 cm in men and ≥0.85 cm in women were considered to be as risky.

The IRLSSG rating scale, which consists of 10 questions and is scored between 0 and 40, was applied to the case group, and the patients were divided into four groups according to the severity of RLS (0–10: mild, 11–20: moderate, 21–30: severe, 31–40: very severe). Then, the International Physical Activity Questionnaire‐Short Form (IPAQ‐SF) was applied to all participants. IPAQ‐SF is a questionnaire consisting of 7 questions in total and evaluating physical activity in the last 7 days according to 4 intensity levels (vigorous‐intensity, moderate‐intensity, walking, and sitting). The form estimates how many days and how many minutes per day people have been physically active in the last 7 days. The metabolic equivalent task (MET) score is calculated from the total physical active time obtained as a result of the test, according to the formula published by Ainsworth et al. (2000). The MET coefficients of walking, moderate‐activity, and vigorous‐activity in IPAQ‐SF are 3.3, 4, and 8, respectively. A result is obtained by multiplying the number of minutes and days of total physical activity by the MET coefficient. Those who score less than 1500 MET‐min/week are defined as physically inactive (sedentary) (Ainsworth et al., 2000; Zhang et al., 2020).

In this study, serum irisin level, complete blood count, ferritin, and serum iron levels were studied in all participants. For Irisin study, venous blood was taken from the participants in the morning after 12 h of night fasting. In addition, it was instructed to the participants that they should not do physical activity before the blood sampling. Relevant blood samples were centrifuged at 4000 rpm for 10 min; then their serums were separated and stored at −80°C until the study day. All samples were thawed at the same time on the study day. Serum irisin levels were studied according to the manufacturer's instructions and standards using the enzyme‐linked immunosorbent assay method with a commercially available kit. Within‐assay and between‐test confidence intervals were <10% and <12%, respectively.

In the descriptive statistics of the data, mean, standard deviation, median lowest, highest, frequency, and ratio values were used. The distribution of variables was measured with the Kolmogorov–Smirnov test. Independent sample t‐test and Mann–Whitney *U*‐test were used in the analysis of independent quantitative data. The chi‐square test was used in the study of independent qualitative data, and the Fischer test was used when the chi‐square test conditions were not met. Pearson and Spearman correlation analyses were used in the correlation analysis. The effect level and cut‐off value were investigated with the receiver operating characteristic curve. SPSS 22.0 program was used in the study.

## 3 RESULTS

The ages of 70 participants included in the study ranged from 27 to 61, and the mean age was 44.1 ± 8.4. Of all participants, 48 (68.6%) were female, and 22 (31.4%) were male. The height, body weight, BMI, waist and hip circumference, and waist/hip ratio values of the patient and control groups included in the study are given in Table 1. Again, the occupational and educational information of the participants and their smoking rates are summarized in Table 1. In the case group, the duration of RLS diagnosis ranged from 0 to 10 years, and the mean diagnosis time was 3.1 ± 2.5 years. Considering the severity of RLS evaluated according to the IRLSSG rating scale, the scores of the case group ranged from 10 to 37, and the mean score was found to be 25 ± 7. The participants’ total physical activity time calculated according to IPAQ‐SF ranged from 271 to 4111 min, and the mean physical activity duration was 1291 ± 872 min. When the serum irisin levels of the participants were examined, they ranged from 3.1 to 54.5 ng/mL, and the mean serum irisin level was found to be 11 ± 12.2 ng/mL (Table 1).

**TABLE 1:** Sociodemographic and anthropometric data of the participants, duration of restless legs syndrome (RLS) diagnosis, severity of RLS, physical activity scale scores, and serum irisin and lipid profile values.

		Min–max	Median	Avg. ± s.d./*n* (%)
Age		27.0–61.0	45.5	44.1 ± 8.4
Gender	Female			48 (68.6%)
	Male			22 (31.4%)
Height (cm)		147.0–185.0	162.5	163.4 ± 8.9
Body weights (kg)		46.0–119.0	75.5	75.4 ± 14.1
BMI		15.5–47.5	27.3	28.2 ± 5.6
Waist circumference (cm)		53.0–130.0	96.0	95.6 ± 13.5
Hip circumference (cm)		86.0–149.0	109.0	109.0 ± 10.5
Waist/hip ratio		0.6–1.1	0.9	0.9 ± 0.1
**Occupation**				
Never worked				30 (42.9%)
Actively working				27 (38.6%)
Retired				13 (18.6%)
**Education**				
Primary school				29 (41.4%)
Secondary school				9 (12.9%)
High school				21 (30.0%)
University				11 (15.7%)
**Smoking**				
Never smoked				45 (64.3%)
Ex‐smoker				12 (17.1%)
Smoking				13 (18.6%)
RLS diagnosis time (year)		0.0–10.0	2.0	3.1 ± 2.5
RLS severity		10.0–37.0	25.0	25.0 ± 7.0
IPAQ‐SF (min)		271–4111	992	1291 ± 872
Irisin (ng/mL)		3.1–54.5	5.4	11.0 ± 12.2
Trigliserit (mg/dL)		34.0–278.0	111.0	129.2 ± 64.0
Total cholesterol (mg/dL)		132.0–292.0	198.0	201.2 ± 35.9
HDLc mg/dL		24.0–71.0	46.0	47.4 ± 10.8
LDLc (mg/dL)		65.0–224.0	121.0	126.3 ± 33.2

Abbreviations: BMI, body mass index; IPAQ‐SF, International Physical Activity Questionnaire‐Short Form.

When the control group and case group were compared in terms of demographic data, height, body weight, BMI, waist circumference, hip circumference, waist/hip ratio, and metabolic complication risk by waist circumferences and waist/hip ratio, there was no statistically significant difference between groups (Table 2). The mean IPAQ‐SF (min) in the control group was 1208 ± 792, whereas it was 1374 ± 950 in the case group. In the IPAQ‐SF (min) scores and physical activity intensities, no significant difference was observed between the control and case groups in light, moderate, and high‐intensity activity (Table 3).

**TABLE 2:** Comparison of sociodemographic and anthropometric data of control and case groups.

		Control group		Case group		
		Mean ± s.d./*n* (%)	Median	Mean ± s.d./*n* (%)	Median	*p*
Age		42.8 ± 8.8	42.0	45.4 ± 8.0	46.0	.210 (*t*)
Gender	Female	22 (62.9%)		26 (74.3%)		.303 (*χ* ^2^)
	Male	13 (37.1%)		9 (25.7%)		
Height (cm)		165.1 ± 9.3	166.0	161.8 ± 8.2		.143 (*m*)
Body weight (kg)		75.5 ± 13.3	76.0	75.3 ± 15.0	74.0	.694 (*m*)
BMI		27.8 ± 5.3	27.3	28.6 ± 6.0	27.2	.828 (*m*)
BMI	˂25	9 (25.7%)		9 (25.7%)		.850 (*χ* ^2^)
	25–30	15 (42.9%)		17 (48.6%)		
	≥30	11 (31.4%)		9 (25.7%)		
Waist circumference (cm)		96.4 ± 13.7	101.0	94.7 ± 13.5	94.0	.194 (*m*)
Hip circumference (cm)		109.4 ± 9.0	109.0	108.7 ± 11.9	109.0	.368 (*m*)
Waist/hip ratio		0.9 ± 0.1	0.9	0.9 ± 0.1	0.9	.414 (*m*)
**Metabolic complication risk by waist circumference**						
No risk		7 (20.0%)		7 (20.0%)		.207 (*χ* ^2^)
Risky		5 (14.3%)		11 (31.4%)		
Very risky		23 (65.7%)		17 (48.6%)		
**Metabolic complication risk by waist/hip ratio**						
No		13 (37.1%)		15 (42.9%)		.626 (*χ* ^2^)
Yes		22 (62.9%)		20 (57.1%)		
**Occupation**						
Never worked		16 (45.7%)		14 (40.0%)		.762 (*χ* ^2^)
Actively working		12 (34.3%)		15 (42.9%)		
Retired		7 (20.0%)		6 (17.1%)		
**Education**						
Primary school		15 (42.9%)		14 (40.0%)		.963 (*χ* ^2^)
Secondary school		4 (11.4%)		5 (14.3%)		
High school		11 (31.4%)		10 (28.6%)		
University		5 (14.3%)		6 (17.1%)		
**Smoking**						
Never smoked		23 (65.7%)		22 (62.9%)		.952 (*χ* ^2^)
Ex‐smoker		6 (17.1%)		6 (17.1%)		
Smoking		6 (17.1%)		7 (20.0%)		

*Note*: *m*, Mann–Whitney *U‐*test; *t*, *t* test; *χ* ^2^, chi‐square test.

Abbreviation: BMI, body mass index.

**TABLE 3:** The International Physical Activity Questionnaire‐Short Form (IPAQ‐SF) (min), physical activity intensity, and serum irisin, lipid profile levels of control and case groups.

	Control group		Case group		
	Mean ± s.d./*n* (%)	Median	Mean ± s.d./*n* (%	Median	*p*
IPAQ‐SF (min)	1208 ± 792	967	1374 ± 950	1067	.530 (*m*)
***IPAQ***					
Light activity	9 (25.7%)		7 (20.0%)		.848 (*χ* ^2^)
Moderate activity	24 (68.6%)		26 (74.3%)		
High‐intensity activity	2 (5.7%)		2 (5.7%)		
Ferritin (ng/mL)	49.3 ± 39.1	37	45.3 ± 37.7	38	.607 (*m*)
Iron (μg/dL)	96.3 ± 36.8	85	86.0 ± 35.9	78	.090 (*m*)
Irisin (ng/mL)	5.1 ± 5.9	3.1	16.9 ± 14.1	12.1	**<.001** (*m*)
Trigliserit (mg/dL)	125.5 ± 67.2	100.0	132.9 ± 61.4	122.0	.481 (*m*)
Total cholesterol (mg/dL)	198.8 ± 38.2	197.0	203.6 ± 33.8	199.0	.545 (*m*)
HDLc mg/dL	51.0 ± 10.6	51.0	43.9 ± 9.8	43.0	**.007** (*m*)
LDLc (mg/dL)	129.3 ± 34.7	120.0	123.2 ± 31.8	122.0	.456 (*m*)

*Note*: *m*, Mann–Whitney *U‐*test; *t*, *t* test; *χ* ^2^, chi‐square test.

**FIGURE 1:**
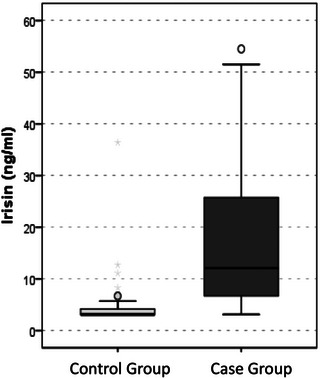
Irisin level was statistically significantly higher in the case group than in the control group.

The mean value of serum irisin level was 16.9 ± 14.1 ng/mL in the case group and 5.1 ± 5.9 ng/mL in the control group, which was statistically quite significant (*p* < .001) (Table 3, Figure 1). When the serum ferritin, iron, triglyceride, total cholesterol, and LDL values in the case and control groups were examined, no significant difference was observed, but the mean HDL value in the case group was 43.9 ± 9.8 mg/dL, whereas it was 51 ± 10.6 mg/dL in the control group, which is statistically quite significant (*p* = .007) was detected (Table 3).

**FIGURE 2:**
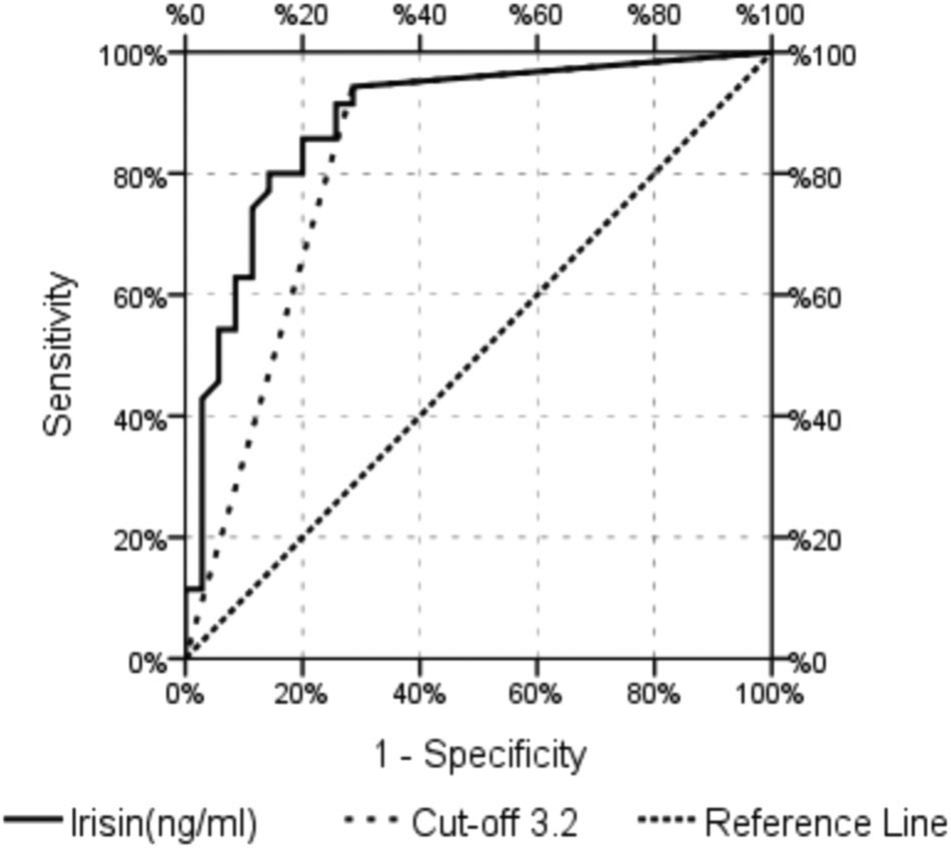
ROC (receiver operating characteristic) curve analysis assesses serum irisin's feasibility as a diagnostic indicator of primary restless legs syndrome (RLS). Serum irisin can discriminate between primary RLS patients and healthy individuals at a cut‐off point of 3.2 ng/mL, with 94.3% sensitivity, and 71.4% specificity.

A significant efficiency [under the curve area 0.886 (0.804–0.967)] of irisin value was observed in the differentiation of patients in the case and control groups (Table 4). The highest value (cut‐off value) of the irisin was 3.2 ng/dL in the differentiation of patients in the case and control groups, and significant efficiency (under the curve area 0.829 [0.726–0.931]) was observed. Sensitivity was 94.3%, positive predictive value was 76.7%, specificity was 71.4%, and negative predictive value was 92.6% (Table 4, Figure 2).

**TABLE 4:** ROC (receiver operating characteristic) curve properties.

	Under the curve area	% 95 Confidence interval	*p*
Irisin (ng/mL)	0.886	0.804–0.967	**<.001**
Cut‐off 3.2	0.829	0.726–0.931	**<.001**
		Sensitivity	94.3%
		Positive predictive value	76.7%
		Specificity	71.4%
		Negative predictive value	92.6%
ROC curve			

## 4 DISCUSSION

The superior result of our study is that serum irisin level was significantly higher in the RLS case group than in the control group. RLS is a common sensorimotor disease affecting sleep quality and quality of life (Harrison et al., 2018). To date, many studies on its pathophysiology have been carried out. Dysfunction of dopaminergic neurons in the spinal cord, genetic factors, and brain iron deficiency is thought to play a role in the pathophysiology (Ainsworth et al., 2000; Allen et al., 2014; Chen et al., 2016; Cheng et al., 2021; Gonzalez‐Latapi & Malkani, 2019; Harrison et al., 2018; Huang et al., 2020; Jodeiri Farshbaf & Alviña, 2021; Lourenco et al., 2019; Manconi et al., 2021; Phillips et al., 2014; Trenkwalder et al., 2018; World Health Organisation (WHO), 2008, Zhang et al., 2020). In a 2021 study, the involvement of the hippocampus and mesolimbic dopaminergic pathway was also pointed up in RLS patients (Mogavero et al., 2021). Relief of symptoms with movement in the clinical diagnosis of RLS suggests that exercise‐related mediators may also play a role in the pathophysiology. Irisin is a polypeptide that release is increased by exercise. Recent studies have shown that irisin is not only found in serum but also crosses the blood–brain barrier and increases the release of BDNF and other neuroprotective agents, especially in the hippocampus. For this reason, studies investigating the effects of irisin on cognitive functions and neurodegenerative diseases have been carried out in recent years (Jodeiri Farshbaf & Alviña, 2021; Lourenco et al., 2019; Phillips et al., 2014; Trenkwalder et al., 2018).

Since irisin is secreted from the muscle and then goes to the adipose tissue, it has been shown in previous studies that serum irisin levels can be affected by the ratio of muscle and adipose tissue. It has been previously reported that there is a positive correlation between serum irisin levels and body weight, BMI, but no correlation between age and waist/hip ratio and serum irisin levels (Stengel et al., 2013). In another study, it was specified that there was a positive correlation between serum irisin levels and the amount of muscle (Huang et al., 2020). In our study, we evaluated the age, gender, height, body weight, BMI, waist circumference, hip circumference, and waist/hip ratio and we did not find a significant difference between the case and control groups.

Another main factor affecting serum irisin levels is physical activity. In a study, RLS symptom severity and physical activity level were evaluated using the IPAQ and were not found to be significant, but it was still stated that in cases with RLS, those who were more physically active had milder symptoms, less pain, and were less physically restricted (Daniele et al., 2013). In our study, we applied IPAQ‐SF to all participants to question physical activity, and we did not find a significant difference between the case and control groups in minute‐based scores. At the same time, when we grouped them as light, moderate, and high‐intensity physical activity in IPAQ‐SF, we did not see a significant difference between the case and control groups in terms of physical activity intensity.

Irisin is also a mediator involved in cholesterol metabolism. In a study, an inverse relationship was reported between serum irisin levels and triglyceride, total cholesterol, and LDL cholesterol levels (Oelmann et al., 2016). Similarly, a recent study stated that as serum irisin levels increased, total cholesterol levels decreased (Cheng et al., 2021). Previous studies have shown that serum HDL levels increase with physical activity (Palazón‐Bru et al., 2021; Sanllorente et al., 2021). However, IPAQ‐SF is a limited and subjective test for evaluating physical activity. The low serum HDL levels in the case group in our study can also be considered evidence that the physical activity of the case group was not higher than the control group. Therefore, the fact that serum irisin levels were significantly higher in the case group than in the control group is not related to physical activity and muscle‐adipose tissue ratios, suggesting that irisin may directly play a role in the pathophysiology of RLS.

In conclusion, we suggest that Irisin may play a role in the pathophysiology of RLS independently of the intensity and duration of physical activity and anthropometric data. The wide and diverse range of duties of irisin suggests that it may also play a role in central and peripheral nervous system‐related diseases. We believe that irisin, which is secreted from the muscle and has effects on the central nervous system with BDNF, may be associated with the pathogenesis of RLS, a disease in which symptoms are relieved by movement. Our results are also promising in this regard. However, the limited number of patients, the use of only IPAQ‐SF to evaluate physical activity, and the clinical diagnosis of RLS without polysomnography are the limitations of our study. It will be more beneficial to assess the relationship between RLS and irisin with a larger number of multicenter cases and use more objective tests to evaluate physical activity. Nevertheless, we believe that the fact that it is the first study in the literature to investigate the relationship between RLS and irisin and that serum irisin levels are significantly higher in the case group will guide future studies.

### PEER REVIEW

The peer review history for this article is available at https://publons.com/publon/10.1002/brb3.3100.

## Data Availability

The data are not publicly available due to privacy or ethical restrictions; research data are not shared.
